# Bringing MicroRNAs to Light: Methods for MicroRNA Quantification and Visualization in Live Cells

**DOI:** 10.3389/fbioe.2020.619583

**Published:** 2021-01-18

**Authors:** Tarana Siddika, Ilka U. Heinemann

**Affiliations:** Department of Biochemistry, The University of Western Ontario, London, ON, Canada

**Keywords:** microRNA, reporter activity, fluorescence, RT-qPCR, next generation sequencing

## Abstract

MiRNAs are small non-coding RNAs that interact with their target mRNAs for posttranscriptional gene regulation. Finely controlled miRNA biogenesis, target recognition and degradation indicate that maintaining miRNA homeostasis is essential for regulating cell proliferation, growth, differentiation and apoptosis. Increasingly, miRNAs have been recognized as a potential biomarker for disease diagnosis. MiRNAs can be found in blood, plasma, and tissues, and miRNA expression and activity differ in developmental stages, tissues and in response to external stimuli. MiRNA transcripts are matured from pri-miRNA over pre-miRNA to mature miRNA, a process that includes multiple steps and enzymes. Many tools are available to identify and quantify specific miRNAs, ranging from measuring total miRNA, specific miRNA activity, miRNA arrays and miRNA localization. The various miRNA assays differ in accuracy, cost, efficiency and convenience of monitoring miRNA dynamics. To acknowledge the significance and increasing research interest in miRNAs, we summarize the traditional as well as novel methods of miRNA quantification with strengths and limitations of various techniques in biochemical and medical research.

## Introduction

Single-stranded microRNAs (miRNAs) are 21–24 nucleotides (nts) in length and are generated from non-coding RNA precursor transcripts. Mature miRNAs regulate gene expression by silencing messenger RNAs (mRNAs), or inducing mRNA cleavage and degradation (Bartel, 2004, 2009; Cai et al., 2009; Chekulaeva and Filipowicz, 2009). MiRNAs bind to partially complementary sequences of target mRNAs, thereby controlling mRNA stability and initiating translational repression of the target genes (Bartel, 2004; Valinezhad Orang et al., 2014). It became clear that miRNAs control almost every pathway and direct cellular proliferation, differentiation, morphogenesis, and apoptosis. Consequently, they are essential to maintain or determine cellular fate (Carthew, 2006; Cai et al., 2009). MiRNAs are encoded in introns or non-coding RNAs and are transcribed as primary transcripts (pri-miRNA) in the nucleus. Here, pri-miRNAs are cleaved into hairpin shaped precursor-miRNA (pre-miRNA) by the ribonuclease Drosha and the microprocessor complex subunit DGCR8. The resulting pre-miRNAs are exported into the cytoplasm via Exportin-5 and further processed by the endonuclease Dicer and other RNA binding proteins to produce mature miRNAs. Guided by Argonaute (AGO) proteins, mature miRNAs are loaded onto the RNA-induced silencing complex (RISC), to finally bind to the 3′-untranslated region (UTR) of target mRNA to suppress translation (Gebert and MacRae, 2019; Michlewski and Cáceres, 2019). Since the initial discovery of two miRNAs, lin-4 and let-7, over 38,000 miRNAs have been annotated in the miRNA reference repository miRbase [www.miRBase.org (Kozomara et al., 2019)].

While many miRNAs have been predicted computationally, development of miRNA reporters and experimental tools has only recently gained traction. To define the role of miRNAs in the cell, biogenesis, functionality and activity of the miRNA need to be experimentally determined, as well as their interaction with predicted targeting sites (Jin et al., 2013). As the consequence of gene regulation through miRNAs and transcription factors (TFs) are quite similar, identification of their cooperative actions has been researched in embryonic development, muscular development and macrophages differentiation. Yet, the individual contribution of mRNA regulation by canonical mRNA expression and degradation pathways vs. miRNA mediated silencing prove difficult to discern (Arora et al., 2013). There are many compelling reasons for monitoring and quantifying of miRNAs in tissues, cell cultures, and transgenic organisms. A conservative estimate is that more than 60% of mammalian genes are controlled by miRNAs, yet most of the target sites remain unexplored (Friedman et al., 2009). Many of these miRNA imperfectly base pair at the 3′-untranslated region (UTR) of their target mRNA, making predictions of miRNA targets even more difficult. It is, however, clear, that miRNAs negatively regulate protein output, and the dysregulated expression of miRNAs correlates with development of many diseases such as cardiovascular disease, retinal disorders, neurodegenerative diseases, diabetes and cancer (Lv et al., 2008; Gommans and Berezikov, 2012; Xin et al., 2012). Additionally, miRNAs may be cell-specific biomarkers for some human cancer as well as diagnostic and prognostic markers of old or novel therapeutic responses (Bartels and Tsongalis, 2009). Conserved miRNAs and changes in their expression can be used as a predictor for disease outcomes, sex chromosome evolution (Naqvi et al., 2018), T-cell development and neural cell specification (Smirnova et al., 2005; Neilson et al., 2007). MiRNAs are used as biomarkers in disease detection and treatment: changes in miRNAs concentrations in serum are utilized to study the effect of radiation treatments (Fendler et al., 2017), and the expression profile of certain miRNAs allows for a classification of cancer stage (Lu et al., 2005). Finally, miRNA levels are indicative of host cell inflammatory responses against certain viruses (O'Connell et al., 2007).

To investigate above mentioned miRNA concentrations and activity in cells, various miRNA assays have been developed to measure the level of miRNA expression, stability and abundance in cells, tissues and organs (Pritchard et al., 2012). It is challenging to detect and quantify miRNAs because of their short nt sequences, different forms of miRNA precursor transcripts, high copy numbers, variations in unique expression, and the difficulty in designing highly specific oligonucleotides for probe-based assays (Chen et al., 2011). Recent advances in the field have led to the development of a number of miRNA profiling methods. In this review we will discuss the different types of miRNA reporters and the approaches to measure miRNA activity with their advantages and disadvantages to give an overview of the current miRNA toolbox.

## Quantification of Total MiRNA

While it has been shown that not all miRNAs in a cell are utilized for mRNA silencing at a given time, it has generally been assumed that the overall amount of miRNA – as regulated by expression, processing, and degradation - correlates with its biological availability to silence mRNA translation. Thus, many methods rely on the quantification of the overall miRNA amount. In general, we distinguish between hybridization, amplification, sequencing, and enzyme-based methods.

### Hybridization Based Methods

#### Northern Blot

The most established method of miRNA detection is northern blotting, which is now used less frequently owing to the advancement of real-time PCR and microarrays. Nonetheless, Northern Blots are still considered the gold standard, as they detect mature and pre-miRNA without amplification bias and can simultaneously determine the size of the miRNA, including size changes caused by post-transcriptionally added nucleotides and accurate expression levels (Lagos-Quintana et al., 2001; Lee et al., 2002; Schmittgen et al., 2004). The general outline of miRNA northern blotting includes initial polyacrylamide gel electrophoresis of extracted RNAs to isolate fractionated small RNAs, followed by the transfer of the RNAs onto a nylon membrane. After crosslinking, radioactive, fluorescently or digoxygenin labeled probes are hybridized to the target miRNA (Lau et al., 2001; Li and Ruan, 2009). To improve the sensitivity for miRNA detection, chemically changed locked nucleic acid (LNA) induced oligonucleotide probes were developed and led to 10-times higher detection efficiency compared to traditional DNA probes, as well as increasing the thermal stability of probes (Várallyay et al., 2008). LNAs are RNA or DNA sequences with modified nucleotides that contain a methylene bridge in the ribose ring structure which is resistant to nucleases mediated degradation (Frieden et al., 2003; Válóczi et al., 2004; Várallyay et al., 2007). Using water-soluble 1-ethyl-3-(3-dimethylaminopropyl) carbodiimide instead of UV radiation for RNA crosslinking to the membrane greatly reduces the movement of RNAs which is more suitable to complementary binding with nucleic acid probes and elevates the membranes' retention ability to bind larger amount of small RNAs (Pall et al., 2007; Pall and Hamilton, 2008). Finally, the combination of non-radioactive digoxigenin-labeled LNA probes and carbodiimide mediated cross-linking of RNAs enables high-resolution northern blotting as a distinctly useful tool for determining length variation of endogenous miRNA and its precursors, and showed higher reproducibility when comparing the blotting signals with deep-sequencing results (Kim et al., 2010; Koscianska et al., 2011). To overcome the problem of multiplexed blots of target RNAs, both radioactive and non-radioactive probes triggered hybridization chain reaction (HCR) can be operated independently for multiplex banding of target miRNA (Schwarzkopf and Pierce, 2016).

##### Strengths and Limitations

Northern blotting is a versatile tool to detect precursors and mature miRNAs with comparatively cheap and non-specialized equipment, but is nonetheless time consuming, and throughput and sensitivity is relatively low (Pritchard et al., 2012).

#### Microarrays

**Figure 1:**
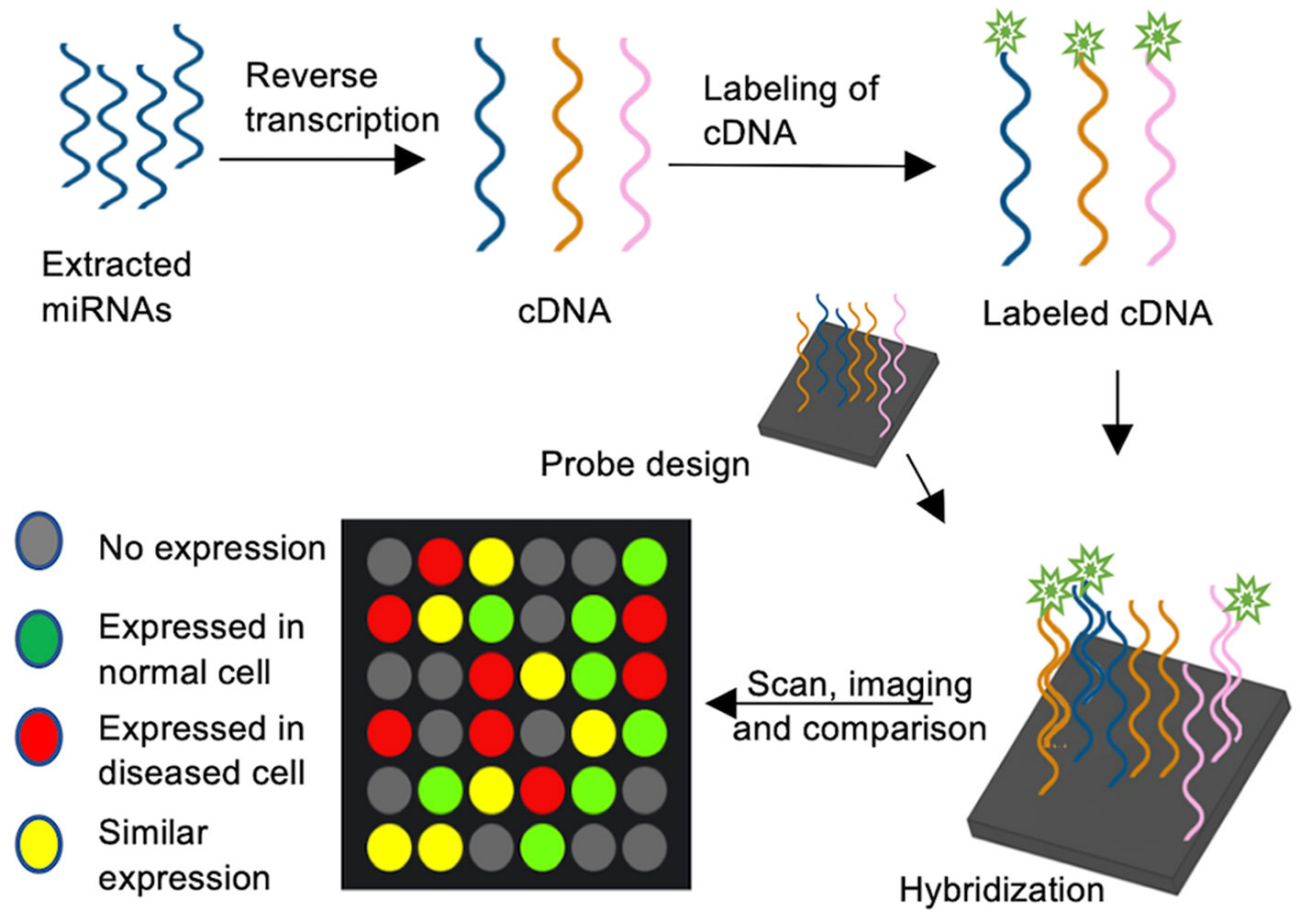
MiRNA Microarrays. cDNA is synthesized by reverse transcription of extracted miRNAs and the cDNAs are subsequently labeled with e.g., a fluorophore tag. Oligonucleotide probes complementary to target miRNAs are spotted on a carrier plate and the fluorescently labeled cDNA is hybridized. The signal intensity corresponds to miRNA abundance.

Microarrays are a universal analysis tool for profiling miRNA expression and are widely used to compare expression profiles. For example, the link between specific miRNAs to cancer cell proliferation and tumorigenesis has been investigated using arrays (Wang Y. W. et al., 2018; Wang Z. et al., 2018). Microarrays can assess the expression patterns of hundreds of miRNA genes in a single assay. They usually are solid surface slides with hundreds of probes spotted in a grid, each specific to a single miRNA gene. Generally, miRNA arrays encompass several major steps (Figure 1): purification of mature miRNA, reverse transcription and simultaneous labeling of the cDNA, and hybridization of the cDNA to high-affinity probes on a glass plate. Depending on the labeling method (biotin, radioactivity, fluorophores) the hybridized arrays are scanned for signals, allowing for quantification and normalization of the data to a control experiment (Zhao et al., 2006; Yin et al., 2008). Microarrays for miRNAs were utilized by Krichevsky et al., where a nylon membrane oligonucleotide array was incubated with radiolabeled cDNA to identify regulatory miRNAs in the mammalian brain (Krichevsky et al., 2003). Nowadays, radioactive labeling is largely avoided and oligonucleotide microchip based arrays on glass slides are coupled with 5′ biotin labeled cDNA probes, allowing for a robust miRnome analysis (Liu et al., 2004). Direct labeling of RNA through various types of fluorescence probes was successfully used to interrogate the role of non-coding miRNA in microarrays (Kampa et al., 2004; Liang et al., 2005). Importantly, miRNA array analysis must include post-data analysis steps, such as normalization and statistical analysis, and often requires data verification with alternate methods, such as reverse transcription quantitative PCR (RT-qPCR). Based on miRNA databases such as miRbase (Kozomara et al., 2019), several companies designed miRNA microarray platforms with specific probes, fluorescent dyes and hybridization steps (Callari et al., 2012), making microarrays a readily available tool and allow for the high throughput quantification of miRNAs. Several studies compared the available platform and analyzed inter-platform reproducibility, finding a poor overlap in miRNA determined to differentially expressed, but a general overlap was found in miRNAs considered to be enriched (Callari et al., 2012; Chatterjee et al., 2015; Franco et al., 2016). Therefore, the experimental approach (pooled biological samples *vs*. true triplicates, RNA extraction methods, etc.) and microarray platform need to be carefully chosen, and enrichment of miRNAs should be verified by an alternate method, such as RT-qPCR.

##### Strengths and Limitations

Microarrays can compare levels of miRNA expression in different organs, tissues and species, and are a major, high throughput tool to analyze hundreds of miRNAs simultaneously. Sensitivity of microarrays is relatively low, mostly due to the amount of miRNA applied to the array. Furthermore, specificity can be compromised in miRNAs with high sequence similarity due to the nature of miRNAs short hybridization sequences and low copy number. Finally, while many miRNAs can be analyzed simultaneously, microarrays do not allow for the discovery of new miRNAs and the cost is comparatively high.

#### Bead-Array Based Profiling

MiRNAs often only differ by a single nucleotide, posing significant challenges due to cross-hybridization of closely related miRNAs in microarrays. This led to the development of bead-based miRNA profiling. Bead-based profiling requires an initial adapter ligation step to the RNA 3′ and 5′ end. Oligonucleotide linkers are ligated at the 3′ and 5′ termini of miRNAs to maintain uniform lengths, avoiding amplification bias of to heterogeneously elongated miRNAs. A biotin tag is introduced during reverse transcription (Miska et al., 2004). Oligonucleotide probes complementary to the miRNAs are ligated to polystyrene beads. miRNAs are sequence specifically labeled with different dyes. The biotinylated cDNAs hybridize to the capture probes, unhybridized probes are washed away, and subsequently analyzed by flow cytometry to denote miRNA identity and abundance as determined by bead color and intensity (Lu et al., 2005).

##### Strengths and Limitations

Bead-based flow cytometry arrays are lower in cost compared to microarrays, involve fewer processing steps and are medium throughput, but require specialized equipment for flow cytometry.

### Amplification Based Methods

#### RT-qPCR

**Figure 2:**
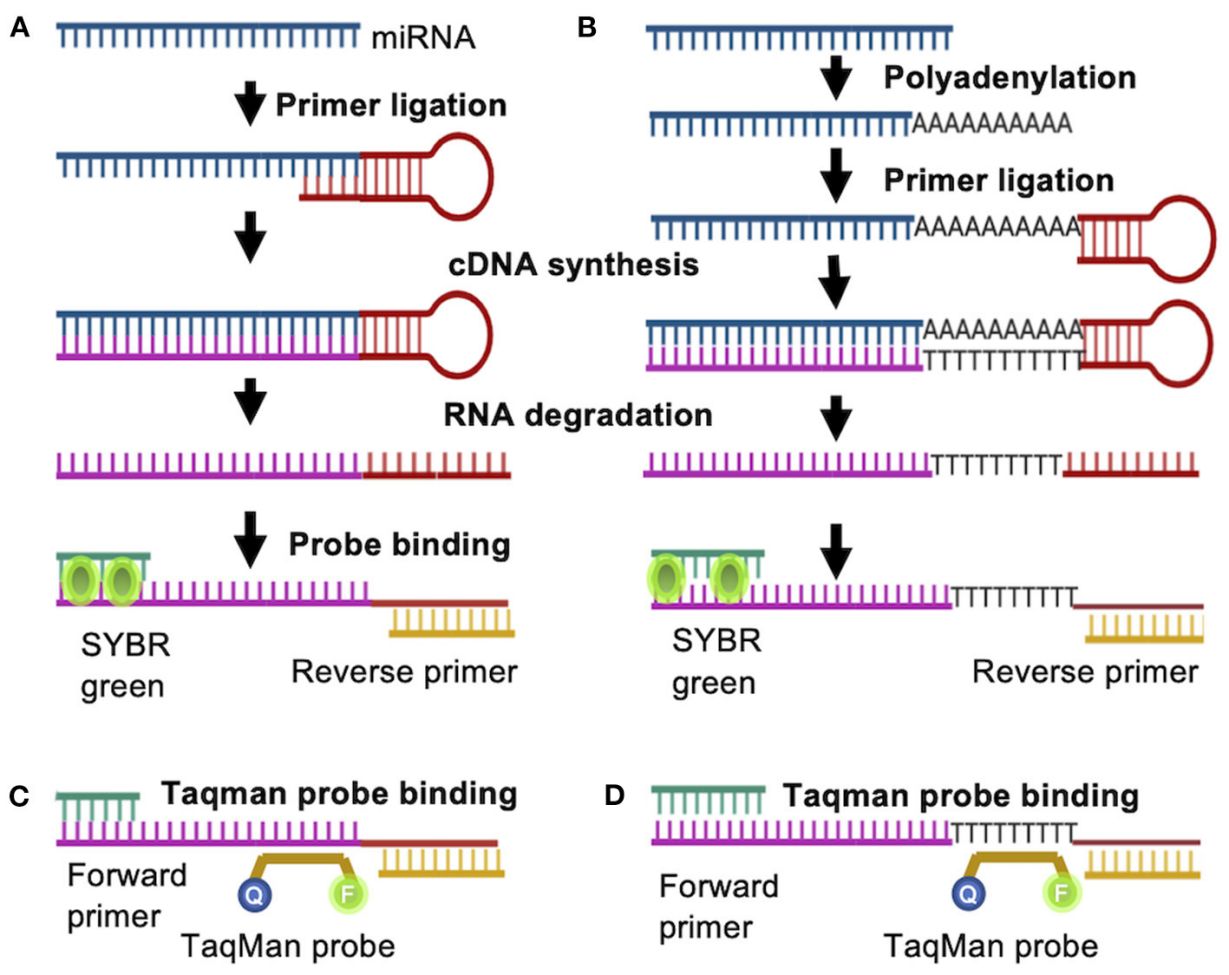
RT-PCR based quantification of miRNA. A Stem-loop RT primer binds to the target miRNA either **(A)** directly or **(B)** indirectly after addition of adenine nucleotides to the 3′- end, allowing for reverse transcription. The generated cDNA is amplified by PCR with a miRNA specific forward primer and a universal reverse primer. Instead of SYBR green, a gene specific **(C)** or universal **(D)** Taqman probe can be used.

To study miRNA and its functions, PCR- based methods generate quantitative data with efficiency, specificity and sensitivity, quickly revealing RNA expression variance between samples (Salone and Rederstorff, 2015). PCR based amplification of miRNA poses some challenges in specificity due to the short transcript lengths. Initially, conventional qPCR with a linear primer followed by poly (A) tailing was used to detect and quantify miRNA but was promptly replaced with RT-qPCR (Chen et al., 2005). A stem-loop RT-based assay (Figure 2) is the most commonly applied method for mature miRNA detection and can discriminate between miRNA species varying in as little as a single nucleotide. Here, the target miRNA is reverse transcribed by annealing a stem-loop primer to the 3′ end of the miRNA for reverse transcription, which is then used as template to amplify the miRNA with a specific forward primer and a universal reverse primer or TaqMan probe (Figure 2A) (Raymond et al., 2005; Hurley et al., 2012; Salone and Rederstorff, 2015). This specific preamplification is used by several RT-qPCR platforms, namely TaqMan array cards or open array cards (Liu et al., 2010). Stem-loop primer based RT-qPCR can be utilized to analyze the expression of one or a few miRNAs using gene specific primers, or be applied in high throughput analysis by utilizing universal stem-loop primers (Yang et al., 2014). The now widely used stem-loop method significantly enhanced specificity and reproducibility (Luo et al., 2012; Mohammadi-Yeganeh et al., 2013).

SYBR green is commonly applied to visualize the formation of double stranded DNA and is often used in RT-qPCR applications. In solution, SYBR green exhibits low fluorescence, but once it binds to double stranded DNA, fluorescence is enhanced, making it an excellent reporter for double stranded DNA concentrations (Figures 2A,B). However, SYBR green dye can be somewhat inaccurate due to double stranded DNA contamination or primer dimers (Bustin, 2000). Alternatively, gene specific Taqman probes can be utilized to detect amplification rates. In this case, the probe is complementary to a DNA strand and is labeled with a fluorophore and a quencher. During amplification, the probe is displaced from the DNA strand, resulting in cleavage of the fluorophore and an increase in the fluorescence signal (Figures 2C,D). A modification of the stem loop primer (Figures 2A,C) approach includes an additional polyadenylation step prior to ligation of the stem loop primer (Figures 2B,D) (Luo et al., 2012; Mohammadi-Yeganeh et al., 2013). This allows for the utilization of universal poly(dT) probe, reducing reagent costs by more than 10-fold when a Taqman probe is utilized (Figure 2D) (Goldrick et al., 2013). This poly(T) containing primer mediated RT-qPCR method was successfully optimized for the analysis of circulating miRNAs as biomarkers for disease (Niu et al., 2015, 2019). RT-qPCR is a reliable, reproducible and highly quantitative method to detect even low levels of RNA and has been applied on the single cell level (Kang et al., 2012).

##### Strengths and Limitations

Depending on the target miRNAs, RT-qPCR is considered a low to medium throughput method, suitable for the targeted quantification of miRNAs. Variation in primer design, and inconsistent data analysis and normalization can negatively affect the reproducibility of RT-qPCR. Nonetheless, due to its high sensitivity and specificity, RT-qPCR is the current gold standard method to verify data obtained by microarrays or next generation sequencing approaches.

#### Amplification Assays

Different nucleic acid sequence-based amplification strategies, for example rolling circle amplification (RCA), hybridization chain reaction (HCR), strand displacement amplification (SDA), loop-mediated isothermal amplification (LAMP) and exponential isothermal amplification assay (EXPAR) have been utilized to analyze miRNAs (Xu et al., 2019). These strategies are constantly improved and modified to increase their analytical potential and we will only briefly introduce these methods, as they have recently been reviewed in detail (Reid et al., 2018). For example, a palindromic padlock probe was employed in RCA to detect let-7a in HeLa cells (Xu et al., 2019), or the application of an additional primer resulted in a branched RCA, that quantifies let-7 variants in single cells with high sensitivity (Cheng et al., 2009). EXPAR is a highly sensitive amplification method that detects miRNA at a constant temperature. The amplification template has two repeated flanking sequences at the 3′ and 5′ terminus, which are complementary to the desired miRNA and separated by a recognition site for nicking enzyme. Target miRNAs are hybridized with the complementary sequences to the template to add nucleotides using a DNA polymerase. Extended sequences containing a nicking endonuclease recognition site are cut by a nicking enzyme to release a sequence which triggers another amplification and is subsequently detected by SYBR green (Jia et al., 2010). SDA and modifications of the initiator primers in LAMP also lead to exponential amplification of the target miRNAs, generating fluorescence allowing quantification of miRNAs. In HCR, two hairpin DNA sequences carry out a series of multiple chain reactions using linear amplification initiated by the complementary binding of miRNA with a template (Dirks and Pierce, 2004).

##### Strengths and Limitations

Exponential amplification is gaining popularity due to the simplicity of procedures without the need of complex equipment, but these methods have proven less accurate in the identification of weakly expressed miRNA (Moody et al., 2017).

#### Next-Generation Sequencing

Next-generation sequencing (NGS) is not only able to profile known miRNAs expression but also suitable to identify various unknown miRNAs variants, which are not recuperated by the conventional targeted methods such as RT-qPCR and microarrays (Willenbrock et al., 2009; Liu et al., 2011). Applications of NGS in miRNA sequencing have evolved rapidly over the past few years. The basic procedure of miRNA sequencing is similar to DNA sequencing, with additional steps to account for RNA library generation. An initial step usually requires the enrichment of small RNAs, which are ligated to 3′ and 5′ adaptors simultaneously. Total small cDNA is generated by reverse transcription followed by amplification and subsequent sequencing (Bar et al., 2008; Hu et al., 2017). Next-generation RNA sequencing generates millions of reads and after raw data processing the expression of thousands of miRNAs can be quantified in one experiment, making NGS a powerful tool in identifying new and known miRNAs and quantifying miRNA expression levels (Metpally et al., 2013). The depth of NGS miRNA sequencing furthermore enabled the identification of new extracellular small RNAs from the blood and cerebrospinal fluid (Burgos et al., 2013), miRNAs associated with fibrosis progression in hepatitis C patients (Van Keuren-Jensen et al., 2016), circulating miRNAs as biomarkers of cervical cancers (Lv et al., 2021), and hippocampal miRNAs associated with memory formation (Tamming et al., 2020), demonstrating the power of NGS in discovery-driven research.

While acquisition of NGS data requires careful sample preparation and specialized machinery, it also requires computational infrastructure for data analysis. Bioinformatics tools are frequently optimized to analyze sequences and data is stored and analyzed in miRNA databases, including sequence reading platforms- Illumina (Hammond et al., 2012), Roche 454 (Johansen et al., 2011), SOLiD (Li et al., 2011), and Ion Torrent (Merriman et al., 2012). Alignment software includes SeqMap (Jiang and Wong, 2008), TopHat (Trapnell et al., 2009), Bowtie (Langmead et al., 2009), MAQ (Li et al., 2008), and megablast (Zhang et al., 2000). Processed sequences are analyzed by several web servers and programs notably including miRExpress, miRanda, miRDeep, DIANA microT, and PicTar (Motameny et al., 2010). While miRNA sequencing will surely gain even more attention as methods are being developed for single cell miRNA sequencing (Faridani et al., 2016), NGS of miRNAs already identified potential biomarkers for laryngeal cancer (Huang et al., 2018). Dysregulated expression of miRNA in inflammation and immune response (Zhang et al., 2019) and differentially expressed miRNAs in kidney disease act as new molecular targets, and NGS data is consistent to validated results of real-time PCR (Liu et al., 2019). NGS sequencing allows for massively parallel analysis and can capture the entire miRnome in a single experiment. NGS has resulted in the identification of previously unknown miRNAs and significantly expanded our knowledge on new miRNAs, that are now annotated in Database repositories [reviewed in Vlachos and Hatzigeorgiou, 2013; Aghaee-Bakhtiari, 2018].

##### Strengths and Limitations

Although NGS is more cost efficient these days, NGS remains expensive and requires extensive computational analysis, which may limit its applications. Nonetheless, the unbiased data acquisition, sequence coverage and depths of NGS is unparalleled by any other available method, and the only discovery-based approach allowing for the identification of novel miRNAs.

### Enzyme Based Assays

#### Invader Assay

**Figure 3:**
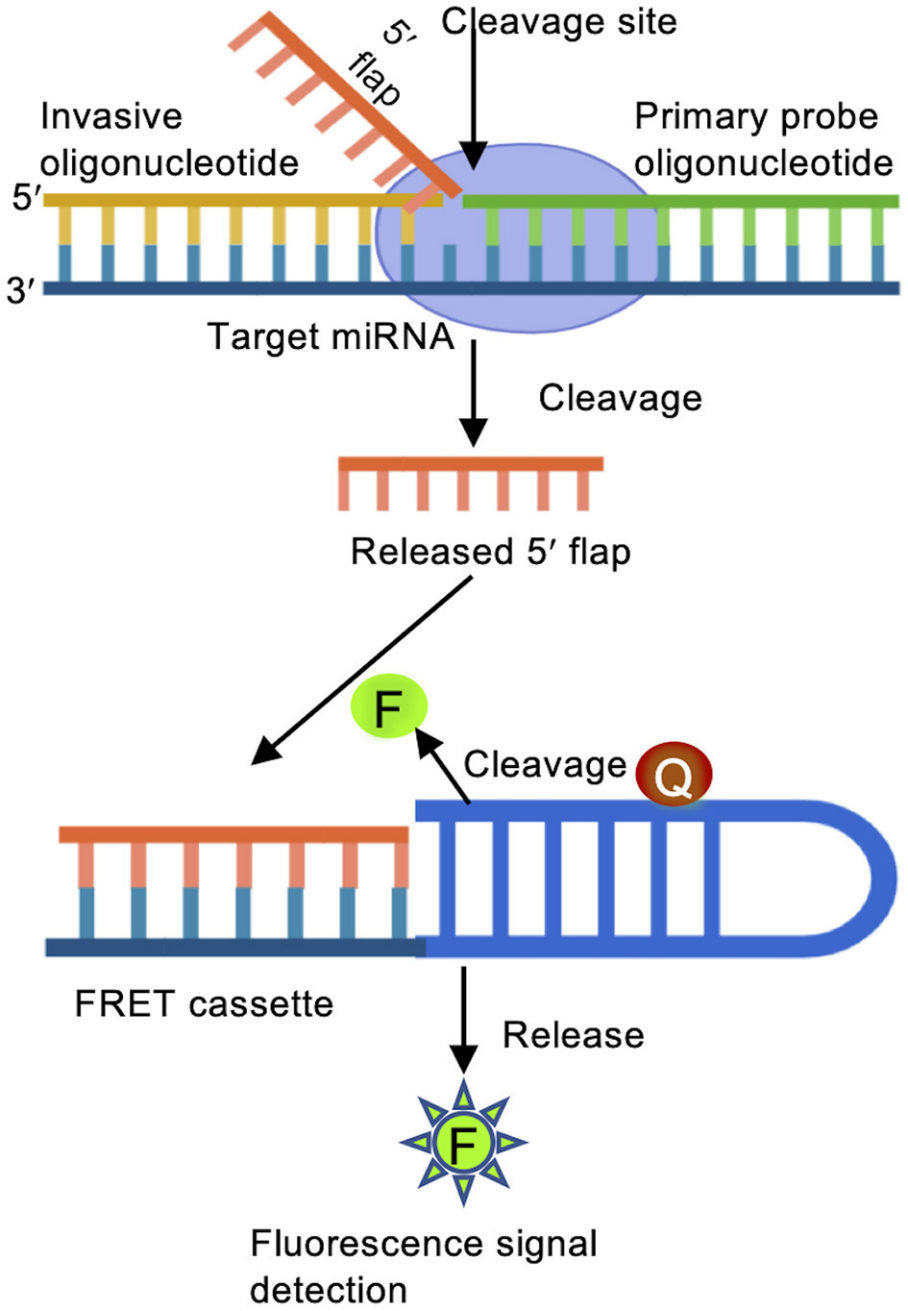
Invader assay. Binding of a primary probe and an over lapping invader oligonucleotide to the target miRNA initiates a cleavage reaction carried out by structure-specific 5′ flap nuclease to release the 5′ flap of the primary oligonucleotide probe. The free 5′ flap acts as an invader oligonucleotide for a second reaction to bind to a FRET cassette containing fluorescent (F) and quencher (Q) molecules. When the 2nd structure is recognized, the FRET cassette is cleaved, emitting detectable fluorescence signals.

The Invader assay is structure-specific enzymatic reaction followed by fluorescence resonance energy transfer (FRET) mediated identification, and offers rapid and specific quantification of miRNA (Allawi et al., 2004). An oligonucleotide probe and an overlapping invasive oligonucleotide are annealed to the target miRNA, and subsequently recognized by an endonuclease that causes the release of 5′ oligonucleotide flaps (Figure 3). Each short flap initiates a second cleavage reaction on multiple matching FRET cassettes containing a fluorescence dye and a quencher. FRET cassettes generate thousands of fluorescence signals within a short period of time. A so-called arrester oligonucleotide is added to hybridize with un-cleaved probes, allowing for a quantification of miRNAs based on the 5′ flaps (Allawi et al., 2004; Olivier, 2005). Mass spectrometry or fluorescence polarization probes can be used for identifying cleaved oligonucleotides.

##### Strengths and Limitations

The invader miRNA assay can identify single nucleotide variance as well as mature and pre-miRNA in parallel in multiple samples and provide medium-throughput results with generalized equipment (Olivier, 2005).

## Quantification of Active MiRNA: MiRNA Activity Reporters

While total miRNA quantification is thought to provide an approximation of active miRNA, recent studies show that miRNAs can be silenced by mono nucleotide addition, or bound to proteins, making them unavailable for post-transcriptional suppression of mRNAs (Chung et al., 2017). These changes in miRNA sequence or availability are not captured in amplification, sequencing or hybridization-based assays. MiRNA activity reporters provide a more accurate snapshot of the amount of active miRNA, in some cases without lysis of cells, allowing for a time resolved or *in-patient* assessment of miRNA activity. Fluorescence or bioluminescence based optical imaging, magnetic resonance imaging (MRI) and positron emission tomography (PET) provide more or less long-time live cell monitoring of miRNA activity (Oh and Do Won Hwang, 2013).

### Luciferase and GFP Based MiRNA Reporters

#### MiRNA Transcription and Target Site Reporters

**Figure 4:**
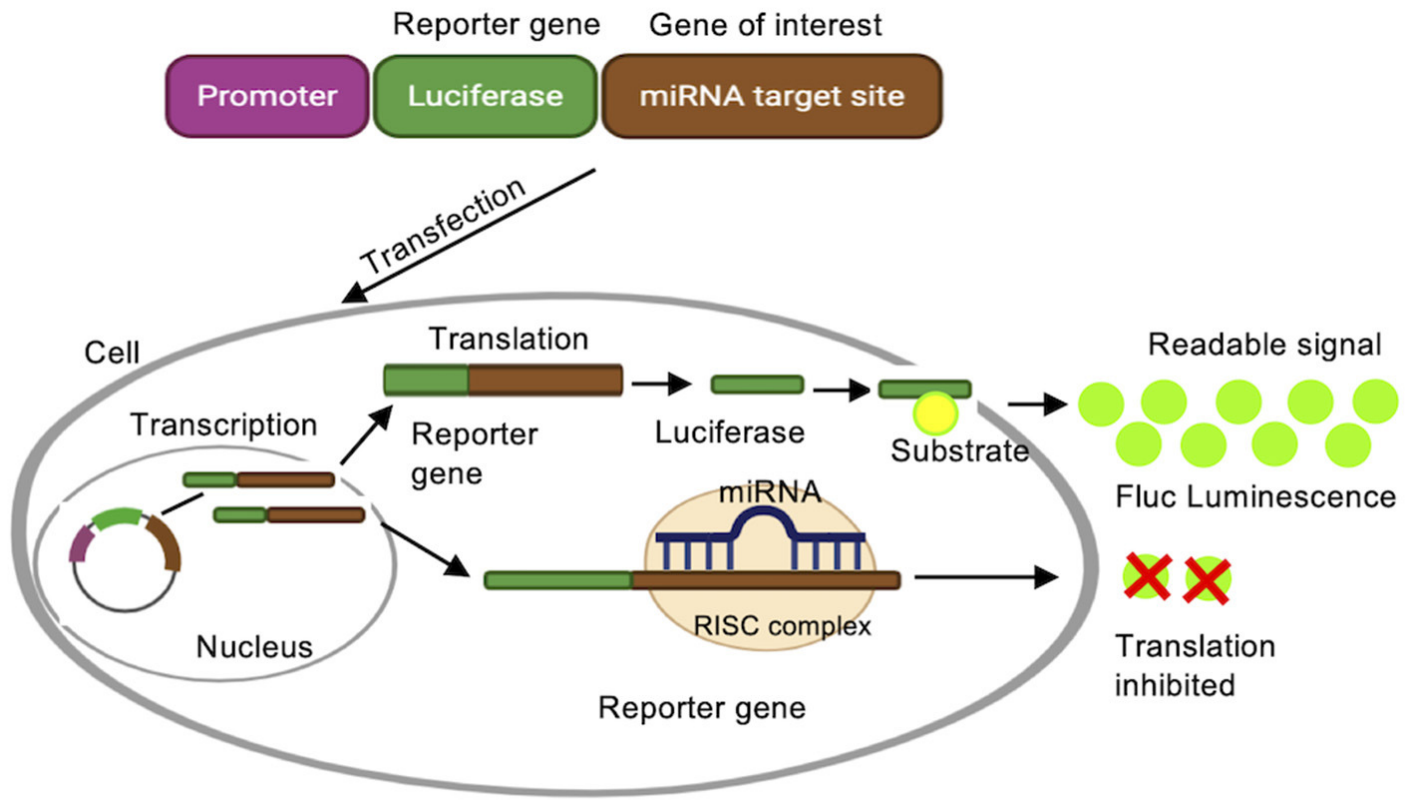
Luciferase reporter assay. A luciferase reporter gene is fused to the promoter sequence of a gene of interest, e.g., the promoter for a specific miRNA, or a target mRNA. Luciferase is transcribed when the native promoter is active, and activity of the reporter protein is detected by converting luciferin substrate to a detectable bioluminescence.

The application of luciferase assays in miRNA research is 2-fold. On the one hand, miRNA expression can be investigated by fusing miRNA promoter to the reporter system. On the other hand, miRNA targets can be validated using the same system, by fusing the 3′UTR of a target gene to the reporter. In luciferase based reporter assays, the enzyme luciferase converts the substrate D-luciferin into oxyluciferin, thereby emitting light (Figure 4) (Haugwitz et al., 2008; Oh and Do Won Hwang, 2013). These reporters are used to monitor miRNA transcription and identify miRNA binding sites. Firefly (Fluc), Renilla (Rluc), and Gaussia luciferase (Gluc) are the most commonly used bioluminescent reporters due to their high sensitivity (Tannous et al., 2005; Haugwitz et al., 2008).

To monitor miRNA expression using a luciferase reporter, transcription of miRNAs and their precursors can be surveyed by fusing the promoter controlling specific miRNA expression to reporter genes. For example, both the 3′-end (miR-9) and the 5′-end (miR-9^*^) of the brain-specific miR-9 function biologically in brain development. Their expression was investigated by separately fusing the Fluc and Gluc genes to the upstream promoter regions of the 3′-end of miR-9 and 5′-end of miR-9^*^ in P19 cells. Differential expression patterns of miR-9 and miR-9^*^ were measured during neuronal cells differentiation (Ko et al., 2008).

As mentioned above, luciferase reporters are also utilized to verify predicted miRNA binding sites on miRNA target genes by monitoring both miRNA and target mRNA transcription. To validate miRNA targets, the 3′ UTR of the target mRNA is fused to the luciferase gene and can be probed in the presence (e.g., transfection of additional miRNAs or different growth conditions) and absence of a miRNA. In this case, miRNA binding to the UTR will decrease Luciferase production.

Dual reporter constructs allow for a simultaneous analysis of miRNAs and target mRNA. For example, expression of a Fluc and Rluc dual reporter construct was used to test the interaction between miR-138 with the 3′-UTR of its target *rhoC* mRNA, identifying the miRNA targeting sites in the coding region of the 3′-UTR (Jin et al., 2013). Fluc and Rluc mediated dual-reporter assays were also utilized to identify the multiple roles of miR-29b (Clément et al., 2015), and miR-529b (Moyle et al., 2017). A dual-fluorescence assay allowed for the detection of subtle changes in miRNAs and successfully identified mutations in targets sites of miR-212 in cardiac disorders (Goldoni et al., 2012). Dual reporter systems provide an efficient way to verify miRNA target sites, and gene regulation of miRNA expression. A large number of fluorescent reporters emitting at different wavelengths can be used to localize e.g., transcription sites and are useful molecular imaging tools (Wessels et al., 2010; Wang et al., 2011).

##### Strengths and Limitations

Luciferase-based reporters are exceedingly useful to investigate the expression of miRNAs or their target genes and generally do not require specialized equipment but are, in nature, low throughput and laborious assays. Furthermore, luciferase-based assays require the addition of luciferin, which makes them less suitable for a time resolved analysis of miRNA expression or activity.

#### *In vivo* MiRNA Activity Reporters

**Figure 5:**
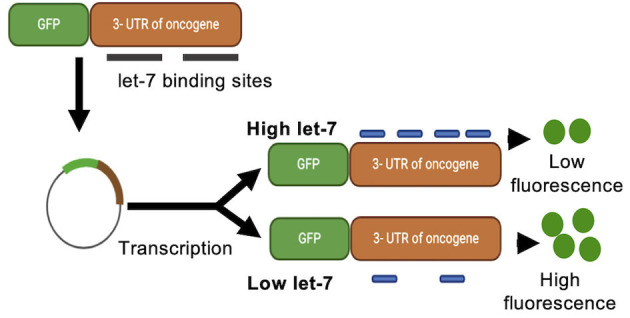
Green fluorescence protein reporter assay. A green fluorescence protein (GFP) reporter gene is fused to a 3′-UTR encoding miRNA binding sites. GFP mRNA translation is controlled by, and responsive to, changes in miRNA activity. Binding of the miRNA to the 3′-UTR of the reporter construct decreases the production of GFP and detectable fluorescence.

We recently developed a reporter gene construct that directly measures the activity on miRNAs over time and during different developmental stages. In these reporter constructs, the green fluorescence protein (GFP) gene is fused to a 3′UTR with specific miRNAs binding sites, making GFP expression responsive to changes in miRNA activity. While luciferase-based assays provide a similar reporter for miRNA activity, in contrast to luciferase expression, GFP expression can be monitored over time, allowing for a time and space resolved analysis of miRNA activity and fewer experimental steps than luciferase-based assays. This reporter was used to successfully measure cellular levels of miRNA let-7a and miR-122 in real-time (Turk et al., 2018). Here, GFP was fused to the 3′-UTR of the oncogene Ras, which encodes let-7a binding sites. GFP fluorescence showed an inverse relationship to let-7a levels (Figure 5). This system allows for the direct measurement of miRNA activity in living cells in a time resolved manner. The GFP reporter system could be expanded in its applications to match the dual reporter assays described above, fusing GFP to the 3′ UTR of a potential target mRNA, while fusing the miRNA promoter to a yellow fluorescent protein (YFP) or red fluorescent protein (RFP), further expanding the toolbox of time resolved miRNA assays.

##### Strengths and Limitations

Similar to the luciferase-based assays, the GFP based assays are low throughput and time intensive, but do not require specialized equipment. Nonetheless, these assays provide a time resolved report on miRNA activity in cell lines, which is not easily accessible with alternate methods.

#### Molecular Beacon (MB) *in vivo* Imaging System

Optical reporter strategies, especially promoter studies, at times cannot differentiate whether the reduced fluorescence signals stem from deregulated gene expression or should be attributed to cellular loss. Plasmid based fluorescence reporters can also usually not be applied as a diagnostic in a living patient. To overcome these limitations, a molecular beacon (MB) *in vivo* imaging system was developed to trace miRNA biogenesis. The MB is a hairpin shaped oligonucleotide structure fused to a fluorescent dye at the 5′ end and a quencher at the 3′ end. In its hairpin form in the absence of miRNA, the fluorescent signal is quenched. Upon binding to the complementary sequence of a target miRNA, the quencher is displaced and a fluorescent signal emitted. MB-based emissions offer excellent tissue penetration and allow for molecular imaging to monitor miRNAs, as has been demonstrated for miR-26a and miR-206 dynamics in muscular cells (Kang et al., 2011). This general method was applied to image miR-124 expression by non-invasive magnetic resonance imaging (MRI). Magnetic nanoparticles conjugated to a fluorescent dye were attached to a double stranded oligonucleotide encoding a miR-124 binding site in one strand and a quencher on the complementary strand, effectively quenching fluorescence in the stem-loop, miRNA free form in the absence of the targets. Upon binding to the miR-124, quencher molecule is dispatched and results in enhancement of fluorescence signal which evaluates the level of miRNA expression. Moreover, magnetic nanoparticles offer *in vivo* cell tracking by imaging miRNA (Hwang et al., 2010; Hernandez et al., 2013). Recently, MRI has been applied to trace circulating miRNAs in serum and allowed for disease prognosis (Regev et al., 2017). Positron emission tomography (PET) imaging was used to localize specific miRNAs using radiolabeled tracer oligonucleotides complementary to the target miRNAs (Mäkilä et al., 2019).

##### Strengths and Limitations

Activity, localization and distribution of miRNA are disease biomarkers (Cheng et al., 2015; Cui et al., 2018), and the above mentioned methods effectively visualize miRNAs in patients as non-invasive imaging systems.

## Conclusion

MiRNAs are differentially expressed, processed and silenced depending on the developmental and physiological context. MiRNAs can have multiple mRNA targets, differ in localization and tissue specific expression and show divergent effects in specific cell lines. The special characteristics of miRNAs, including their small size, high sequence similarity and sometimes low expression, required the development of new, highly sensitive, and specific protocols. MiRNAs in cells can be quantified by the available methods based on their biogenesis, miRNA types and research interest. Unlike intracellular miRNAs, circulating miRNAs are present in the extracellular environment or body fluids and often act as a biomarker for disease such as progression of cancer. Considering the low concentrations and high diversity of extracellular miRNAs, NGS is the preferred method due to its high sensitivity, but microarray and RT-qPCR have also been utilized to detect circulating miRNAs (Cui et al., 2019). In this review, we summarized many of the available methods to quantify miRNA expression, identify new miRNAs and miRNA targets, and measure miRNA activity. The approach of choice often depends on the research question: NGS is unparalleled in its ability to discover new miRNAs, while microarrays are highly useful when comparing the abundance of hundreds of known miRNAs in a more cost-efficient manner. Yet, these methods still depend on RT-qPCR for verification, and neither method illuminates the impact of miRNAs on target mRNAs similarly to luciferase of GFP-based reporter systems, or localize miRNAs in living systems as seen in molecular beacon systems. Thus, any one system is often not sufficient, and a combination of above approaches is usually employed to characterize miRNA functions in depth.

While new methods have been developed to address many of the challenges in miRNA research, the high throughput methods, such as microarrays and NGS remain cost prohibitive to be used on a day to day basis, challenging the science community to come up with novel, more cost-effective methods. Furthermore, the overlap in results from platforms is often lacking, as we outlined for the different microarray platforms. Here, data normalization plays a crucial role in the quantitative miRNA analysis tools. Usually, one or more endogenous control RNAs are used to normalize sample readout in RT-qPCR, microarrays and NGS, assuming that the control RNA does not vary in expression between samples to allow for an adjustment in data variation. While a similar invariance assumption is used in e.g., western blots, where GapdH is often used as a loading control and data is normalized to this protein, the invariance of a control RNA should be verified before designating a certain control RNA as reference. Alternatively, the global mean expression of all miRNAs has been used as a normalizer for microarray and NGS experiments, assuming that while individual miRNAs vary in expression, the overall miRNA content between samples would be similar, but again this may be based on a false assumption. The differences in normalization processes likely account for much or the inconsistency seen between different datasets (Gunaratne et al., 2010; Sales et al., 2010). Finally, RNA extraction and preparation methods, as well as the sample types can significantly impact the results of miRNA profiling, requiring rigorous quality controls. While cost efficiency, sensitivity and standardization remain challenging, the toolbox for miRNA detection, quantification, annotation and analysis are constantly expanding and evolve alongside with new discoveries in the field.

## Author Contributions

Both authors listed have made a substantial, direct and intellectual contribution to the work, and approved it for publication.

## Conflict of Interest

The authors declare that the research was conducted in the absence of any commercial or financial relationships that could be construed as a potential conflict of interest.
